# An Image Classification Method Based on Adaptive Attention Mechanism and Feature Extraction Network

**DOI:** 10.1155/2023/4305594

**Published:** 2023-02-17

**Authors:** Juanjuan Luo, Defa Hu

**Affiliations:** ^1^Hunan International Economics University, Changsha 410205, Hunan, China; ^2^Hunan University of Technology and Business, Changsha 410205, Hunan, China

## Abstract

The convolution neural network (CNN) not only has high fault tolerance but also has high computing capacity. The image classification performance of CNN has an important relationship with its network depth. The network depth is deeper, and the fitting ability of CNN is stronger. However, a further increase in the depth of CNN will not improve the accuracy of the network but will produce higher training errors, which will reduce the image classification performance of CNN. In order to solve the above problems, this paper proposes a feature extraction network, AA-ResNet with an adaptive attention mechanism. The residual module of the adaptive attention mechanism is embedded for image classification. It consists of a feature extraction network guided by the pattern, a generator trained in advance, and a complementary network. The feature extraction network guided by the pattern is used to extract different levels of features to describe different aspects of an image. The design of the model effectively uses the image information of the whole level and the local level, and the feature representation ability is enhanced. The whole model is trained as a loss function, which is about a multitask problem and has a specially designed classification, which helps to reduce overfitting and make the model focus on easily confused categories. The experimental results show that the method in this paper performs well in image classification for the relatively simple Cifar-10 dataset, the moderately difficult Caltech-101 dataset, and the Caltech-256 dataset with large differences in object size and location. The fitting speed and accuracy are high.

## 1. Introduction

Image classification is an image processing technology that extracts effective features from the most original image data and then achieves the goal of distinction of different categories of objects in the image. Now, image classification is one of the most important research hotspots in the field of computer vision. To realize image classification with traditional methods, it must be realized in two stages. First, the unstructured data of the original image data must be converted into structured data used to represent features. Then, the structured feature data must be input into a trainable classifier. Finally, the classification results can be obtained [1, 2]. However, the traditional image classification methods have some shortcomings in that the accuracy of image classification depends on the effectiveness of feature extraction to a large extent [3]. Sometimes, there are some features without discrimination in the image that may have a great negative impact on the classification results. However, the method based on CNN does not need complicated feature extraction and can directly integrate the feature analysis of image data into the CNN model. Only by adjusting the weight and offset of the network model, the effective distinction of image features can be quickly realized [4, 5].

In traditional methods, image classification often needs to work in stages. The first step is feature learning and then feature coding. There are also spatial constraints, of course, the classifier is indispensable, and the process is complex [6, 7]. This traditional method may be effective for some simple image classification tasks, but if the traditional classification method is applied to the actual complex scenes, the results may not be too optimistic. Therefore, researchers began to try using convolutional neural networks to solve the task of image classification, using nonhuman operation methods to extract features from images and analyze the sample data in some aspects, to achieve the purpose of classifying specific targets into a certain label in a known mixed category [8]. However, when using CNN model for image classification, the image classification performance of CNN has a very important relationship with the depth of the network model. The network depth is deeper, and the fitting ability of the CNN is stronger. However, with the increasing depth of the network model, the classification accuracy of the model is not improved, the gradient disappears, and the network model produces higher errors [9, 10]. In view of the above problems, the ResNet model can solve the problem of performance degradation of the model under deep conditions to a certain extent [11]. Recent research shows that the performance of convolutional neural networks can be improved using cross-layer connections. The typical residual network (ResNet) uses this idea to achieve a very good image recognition effect through the identity mapping method. However, in the residual module, the layout of cross-layer connecting lines does not reach the optimal setting, resulting in the redundancy of information and waste of layers [12, 13].

The image classification performance of CNN has an important relationship with its network depth. The network depth is deeper, and the fitting ability of CNN is stronger. However, a further increase in the depth of the CNN will not improve the accuracy of the network but will produce higher training errors, which will reduce the image classification performance of the CNN. In order to solve the above problems, this paper proposes a feature extraction network, AA-ResNet, with an adaptive attention mechanism. The residual module of the adaptive attention mechanism is embedded for image classification.

## 2. Related Work

In recent years, with the advent of the big data era and the development of computer hardware conditions, people have started a more comprehensive and in-depth study of deep learning. Through a large number of theories and experiments, it is proved that the convolutional neural network in deep learning plays an irreplaceable role in the field of image classification. It can obtain image features through a large number of sample training, and the designed classifier is closely related to the extracted features [14]. Compared with traditional classification algorithms, convolutional neural networks can automatically learn the features of image data, and people do not need to spend a lot of experience manually extracting image features. This has a significant effect on image classification tasks with large sample sizes and small differences between categories [15].

The extremely deep convolutional neural network will not only cause the gradient disappearance but also increase the risk of network overfitting, which will affect the accuracy of image classification of the network model. The initial research method is mainly to solve the problem of network gradient disappearance by initializing the network model and conducting hierarchical training. At present, deep convolution neural networks mostly use the activation function ReLU to alleviate the problem of gradient disappearance. Compared with the sigmoid function, the ReLU function is more effective in alleviating the gradient disappearance [16]. The direct supervision method can be used to train the deep CNN model, but in the process of training the deep CNN model, once some inputs of the activation function ReLU enter the hard saturation region, the corresponding weights cannot be updated quickly [17]. The appearance of the neuron death phenomenon will make CNN difficult to converge. Therefore, many new activation functions have been proposed one after another, for example, the PReLU function introduces additional parameters to improve its performance. Another example is the ELU (exponential linear unit) function, which combines the Sigmoid and ReLU functions. Because of its left side saturation, the gradient disappearance problem of the network model can be greatly alleviated [18, 19]. Another example is the PELU (parametric exponential linear unit) function, which is to add parameters to the ELU function and controls the performance of the activation function by training the network model and updating the parameters of the network model in time to better control the offset drift and gradient disappearance during the training of the network [20, 21].

The depth of the CNN is further increased, but the accuracy of the model is not improved. On the contrary, the phenomenon of the gradient disappearing occurs, which results in a higher error. For the above problems, the ResNet model is better than an ordinary convolutional neural network in terms of convergence and classification performance [22]. The main innovation of the ResNet model is to introduce a residual structure into the network, which can quickly transfer the results of the previous layer to the next layer network. When the network model is further deepened, the error will not continue to increase, which makes the ResNet model have more layers and more accurate accuracy [23, 24]. With the increasing depth of the network model, the accuracy of the network model is gradually far from that of the ordinary network model. Obviously, this solves the problem of degradation of the image classification performance of the network model under the condition of depth to a certain extent [25].

## 3. Feature Extraction Network Based on Selective Attention Mechanism

### 3.1. Detailed Steps of Residual Block

The detailed steps of selecting the residual block based on the attention mechanism can be summarized as follows:

**Step 1 .**

For the intermediate feature map in the neural network layer X_0_ ∈ R^H×W×C^, to reduce the parameters and calculation amount, it is sent to the convolution kernel with a size of 1 × 1 to obtain the feature map X_1_ ∈ R^H×W×C_1_^, where H × W represents the spatial dimension of the corresponding feature map C and C_1_ representing the channel dimension of the corresponding feature map;

**Step 2 .**

Group convolution is used to perform a channel-based grouping operation on the feature map X_1_ to obtain a plurality of subfeature maps with the same dimension, X_1_={x_1_ ⋯ , grop}, x_i_ ∈ R^H×W×C_1_/group^, where group represents the number of subfeature maps, i={1, ⋯, group}, and x_i_ represents the i th sub feature map;

**Step 3 .**

The specific operations of the spatial group enhanced attention transformation adopted for each subfeature map x_i_ are as follows:

1. For all the obtained subfeature maps x_i_ ∈ R^m×c^, the global average pooling operation F_gp_(∙) based on space is performed to obtain the global semantic vector g ∈ R^l×c^ of the subfeature map x_i_, where m=H × W represents the spatial dimension of the subfeature map and c=C_1_/group represents the channel dimension of the subfeature map;
2. Use the global semantic vector of the subfeature map x_i_ and multiply it with each subfeature map point to obtain the importance coefficient c_i_ ∈ R^m×l^ corresponding to each subfeature map;
3. Each importance coefficient c_i_ is normalized in the spatial dimension to obtain c_i_′;
4. Scale and translate each of the standardized importance coefficients c_i_′ to obtain a_i_;
5. The newly generated importance coefficient a_i_ generates a spatial enhanced feature map x_i_′ for each subfeature map through a Sigmoid function *σ*(∙) combined with subfeature maps x_i_ corresponding a_i_;
6. The binding step (5) was formulated as the spatial enhancer feature map x_i_′, resulting in the feature maps X_i_′={x_i_′ ⋯ , group′}, x_i_′ ∈ R^H×W×C_1_/group′^, where group′ represents the number of spatial enhancer feature maps and i={1, ⋯, group′}, and x_i_′ represent the ith post enhancer feature map;
7. Feed X_i_′ into the convolutional layer in which the convolution kernel was 1×1 and perform ascending dimension manipulation, obtaining the feature map X_0_′ ∈ R^H×W×C^ which has the same dimension of X_0_, where H × W representing the spatial dimension of the feature map X_0_′ and C representing the channel dimension of the feature map X_0_′;
8. Formulation of step (7) as an intermediate feature map X_0_ combined with the newly derived feature map X_0_′, thus they yield the output feature map ${\hat{X}}_{0}\in R^{H\times W\times C}$ for this spatially grouped attention module, where H × W represents the spatial dimension of feature Fig. ${\hat{X}}_{0}$ and C represents the channel dimension of the feature map ${\hat{X}}_{0}$;

Intermediate feature maps obtain an enhanced attentional feature map when they go through the residual blocks of the middle layers of the network from the above steps. The feature maps were augmented to different degrees by stacked residual blocks. Similarly, with the aid of groupwise convolution, the width of the network is boosted, further enhancing the semantic information of the feature maps. The classification accuracy of the network model can be greatly improved by the network model [26, 27]. Therefore, this paper chose the ResNet101 residual network model as the benchmark model, and in the next step, attentional mechanisms will be incorporated into the ResNet101 model to further improve the classification accuracy.

### 3.2. Residual Module Improvement

**Figure 1:**
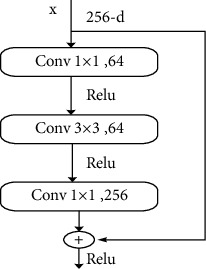
Network model for adding residual blocks.

The residual network can deepen the network without reducing the efficiency of the model, and its schematic is shown in Figure 1.

A neural network with the input of x and the output of *α*^[l]^. Adding new two layers to the end, the output becomes *α*^[l+2]^. These two layers that are newly added are turned into residual blocks. The network is activated using the ReLU activation function, and none of the activation values is less than 0. The value of *α*^[l+2]^ can be represented by (1):

(1) $$\begin{array}{c} \alpha^{\left[l+2\right]}=f\left(z^{\left[l+2\right]}+z^{\left[l\right]}\right). \end{array}$$

Of these, f was the activation function; z^[l+2]^ was the unactivated network output for the layer l+2; *α*^[l]^ was the identity mapping of layers 11 to 22. From (1), it is guaranteed that *α*^[*l*]^ is the same dimension as z^[l+2]^ to be computable. (1) can also be unfolded, as shown in (2):

(2) $$\begin{array}{c} \alpha^{\left[l+2\right]}=f\left(\omega^{\left[l+2\right]}\alpha^{\left[l+2\right]}+b^{\left[l+2\right]}\alpha^{\left[l\right]}\right). \end{array}$$

Even if *ω*^[l+2]^ tends to 0, b^[l+2]^ is equal to 0, *α*^[l+2]^=f(*α*^[l]^), since the entire network uses ReLU activation, *α*^[l+2]^=*α*^[l]^. That is, the jump connection can make it easy for the network to learn *α*^[l+2]^=*α*^[l]^. That is to say, even if the entire network adds two layers, the learning efficiency of the network will not decrease.

**Figure 2:**
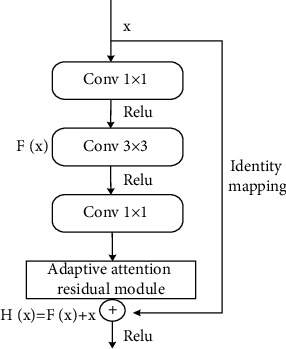
Residual unit integrated into attention mechanism.

It is easy to learn the identity function of the network. Even if there are two more layers, *α*^[l]^ can be assigned to *α*^[l+2]^ through identity mapping. That is, the network performance will not be affected, and the network efficiency can be improved. In practice, *ω*^[l+2]^ will not be 0. That is, new information can be learned through the residual block. Networks are better than just learning identity functions. The ordinary network without residual block is difficult to learn the identity connection, and it will not be better than before. Therefore, creating residual blocks is advantageous to improve network efficiency. The parameters of the network model can be greatly reduced. Its principle is to reduce the dimension first and then increase the dimension by using the convolution kernel of size. In order to better extract the features of the image, the attention mechanism module is integrated into the original residual unit to form a new residual structure. The specific integration position is shown in Figure 2.

Although the attention mechanism will add some computational overhead, it is very beneficial to improve the classification accuracy of the network model. Figure 2 shows a feature map with an input of 64 × 56 × 56, and the calculation process of the attention weight of each channel is generated through the residual unit. The feature map with the input of x and the size of 64 × 56 × 56 will first pass through three two-dimensional convolutions. These two-dimensional convolution layers play a role in reducing the number of parameters. The first two convolution layers are activated by the ReLU function. The left side shows the relevant parameters of the step size, convolution kernel size, and filling value. After three convolutions and two activation operations, the output feature map size is 256 × 56 × 56.

Secondly, when entering the attention mechanism module, the feature map output in the previous step will first undergo adaptive global average pooling to obtain an output feature map with a size of 256 × 1 × 1; after removing one dimension and exchanging the positions of the remaining two dimensions, one-dimensional convolution is performed. In this step, the convolution kernel size is 3, the step size and filling are 1, and the feature map size is 1 × 256. By exchanging and expanding the dimensions of the feature map by one dimension, then Sigmoid activation is performed to obtain 256 weight values representing the weights of each dimension. The 256 weight values are multiplied by the initial feature information to obtain a new output feature map whose size is 256 × 56 × 56. The above is the process of inputting x and obtaining F(x).

Then, identity mapping is performed. In order to ensure that the feature map with input 11 and the feature map with output 22, feature maps have the same dimension and can be added, the dimension of *x* must be increased first, as shown on the right side in Figure 2, so that the channel dimension becomes 256. The output H(x) = F(x) + *x* of the residual unit can be obtained.

## 4. Experimental Comparison and Analysis

### 4.1. The Setting of Model Parameters

When training the AA-ResNet model in this paper, the hyperparameters that need to be set mainly include the size of batch training, the size of the learning rate, the selection of classification number, and the weight decay rate. The size of batch training determines the descending direction of the AA-ResNet model. When the dataset is large enough, the size of batch training should be appropriately reduced to greatly reduce the calculation amount. If the data amount is small and there is noise data, the batch training should be set to a large value to reduce the interference of noise data. When batch training reaches a certain value, the AA-ResNet model is optimal in terms of training time and convergence accuracy.

The magnitude of weight update is closely related to the learning rate, so it is very beneficial to set the learning rate in an appropriate range to reduce the gradient of the AA-ResNet model to the optimal value. If the learning rate is set too large, the weight of the AA-ResNet model will exceed the optimal value and then swing back and forth at the end with a small error. However, if the learning rate is set too small, the optimization of the AA-ResNet model will require a lot of time, and even the model may not converge. The initial learning rate of the AA-ResNet model in this paper is set to 0.1. However, with the increase in the number of iterations of the model, it is gradually adjusted to 1/10000, to improve the accuracy of the AA-ResNet model while obtaining a faster training speed. In the training process, overfitting often occurs. The greater the weight of the AA-ResNet model, the greater the risk of overfitting. In order to reduce the risk of overfitting, a penalty term is added to the error function. The weight decay rate is the main parameter for calculating the regularization of L_2_. The main function of the weight decay rate is to adjust the influence of the complexity of the AA-ResNet model on the loss function. The regularization of L_2_ can obtain parameters with small values to reduce the risk of model overfitting.

### 4.2. Model Evaluation Index

In this paper, the evaluation indicators of the model include training time, train acc, test acc, loss value parameters, drawing confusion matrix, accuracy, and loss value change line graph, where train acc refers to the proportion of the number of correctly predicted samples in the training set to the total number of samples in the training set. In semantic segmentation, it refers to the proportion of correctly predicted pixels in the training set to the total number of pixels. Test acc refers to the proportion of the number of correctly predicted samples in the test set to the total number of samples in the test set. In segmentation, it refers to the proportion of correctly predicted pixels in the test set to the total number of pixels. The loss value reflects the degree to which the predicted value of the model is different from the real value. Calculate the cross entropy loss function of the loss value. For sample *i*, construct a vector y^(i)^ ∈ R^q^ so that the y^(i)^ element (discrete value of the category of sample *i*) is 1 and the rest is 0 to represent the real label; ${\hat{y}}^{\left(i\right)}$ denotes a probability distribution predicted by the model; Θ denotes model parameters. Training time refers to the average time of training an epoch for the training set.

(3) $$\begin{array}{c} H\left(y^{\left(i\right)},{\hat{y}}^{\left(i\right)}\right)=-\overset{q}{\underset{j=1}{\sum }}y^{\left(i\right)}\;\log\;{\hat{y}}^{\left(i\right)}, \end{array}$$

(4) $$\begin{array}{c} l\left(\Theta \right)=\frac{1}{n}\overset{n}{\underset{i=0}{\sum }}H\left(y^{\left(i\right)},{\hat{y}}^{\left(i\right)}\right). \end{array}$$

### 4.3. Data Set

The image classification data used in this experiment include the Cifar-10, Caltech-101, and Caltech-256 data sets. Cifar-10 is a data set containing 60000 images, of which 50000 are training images and the other 10000 are test images. Each photo is a 32*∗*32 pixel color photo. All photos belong to 10 different categories, namely airplane, automobile, bird, cat, deer, dog, frog, horse, ship, and truck. In this experiment, 50000 training images are used to construct the dataset, and 5000 images are used in each class. According to the ratio of 8 : 2, 40000 images are constructed as the training set, and the other 10000 images are constructed as the test set. Caltech-101 is a data set containing 9144 images. The image is a color image, most of which have a separation rate of 300*∗*300 pixels. All the images belong to 101 different categories, including accordion, chair, crab, and laptop. The number of images in each category ranges from 31 to 800. Caltech-256 is a data set containing 30607 images. The image is also a color image. All the images belong to 101 different categories, including cakes, CDs, coins, frogs, grapes, and so on. The number of images in each category ranges from 80 to 827.

### 4.4. Cifar-10 Data Set Experimental Results

The Cifar-10 data set is a relatively easy data set for image classification. One reason is that the number of images available for training in the Cifar-10 data set is huge, with a total of 60000 images, and 50000 images were used in the experiment. Second, there are only ten categories due to fewer categories. According to the theoretical research and inference of existing algorithms, after the training of the convolutional neural network model, higher accuracy can be obtained. This is supported by the data in Table 1.

It can be seen from Table 1 that the accuracy of the five models on the Cifar-10 data set is high and the difference is small. The accuracy of the training set was more than 95%, and the accuracy of the test set was about 90%. From the data, we can find that the model in this paper has the best fitting for the dataset. The accuracy of the training set and the test set is the highest among all models, which are 99.96% and 92.43%, respectively. The loss value was the lowest, only 0.00129.

**Figure 3:**
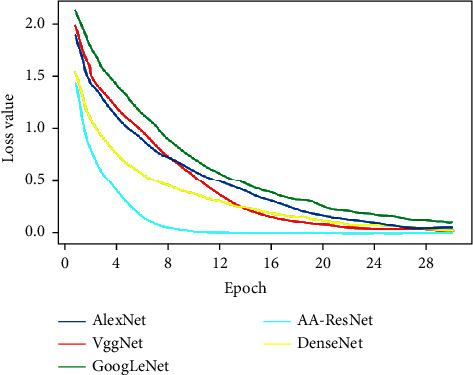
Loss value of Cifar-10 data set.

It can be observed from Figure 3 that the loss values of the five models gradually decrease from the interval of 1.5 to 2 to about 0. Among them, the cyan curve of the AA-ResNet model is located at the bottom as a whole, the loss value is close to 0 at about 8 epochs, and the fitting speed is fast.

**Figure 4:**
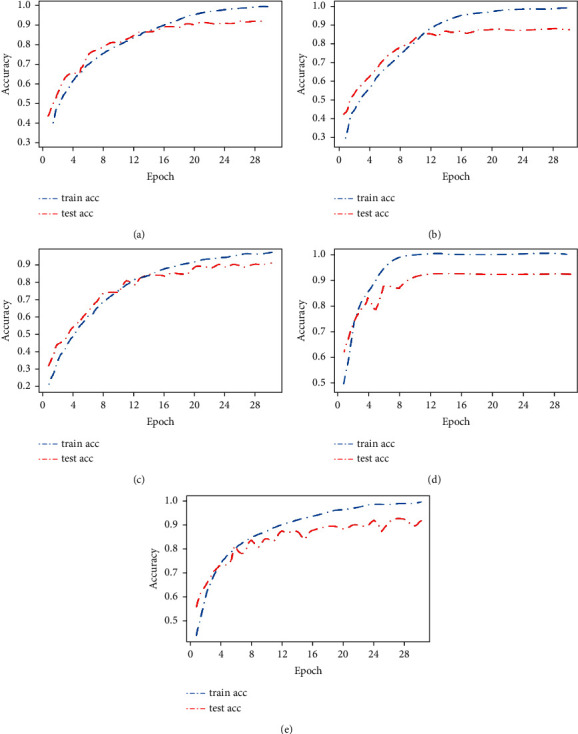
Accuracy rate of Cifar-10 data set. (a) AlexNet. (b) VggNet. (c) GoogLeNet. (d) AA-ResNet. (e) DenseNet.

The accuracy broken line graph in Figure 4 reflects the changes in the training set accuracy rate train acc and the test set accuracy rate test acc as the number of epochs increases. It can be found that the accuracy starting point range of the training set and test set of AlexNet, VggNet, and GoogLeNet models is relatively consistent, between 20% and 40%, while the starting point range of AA-ResNet is relatively high, between 50% and 60%, and the initial fitting is relatively good. At the same time, it can also be seen that the accuracy rate of AA-ResNet has a large initial discount slope, which indicates that the accuracy rate is greatly improved with less epoch numbers, it is also a performance of excellent performance. It was also found that when the accuracy of the training set tends to be stable, the accuracy of the test set does not change.

In terms of data parameters and overall mapping, the classification results of the five models are excellent in the Cifar-10 dataset experiment. This is not only because the classification difficulty of the Cifar-10 data set is low but also reflects the superiority of convolutional neural networks in the field of image classification. However, there are small differences between the models. AA-ResNet has the best performance, high accuracy, and fast fitting speed in the Cifar-10 data set. The comprehensive performance of the other models is relatively consistent.

### 4.5. Caltech-101 Data Set Experimental Results

The classification difficulty of the Caltech-101 data set is significantly higher than that of the Cifar-10 data set. Not only has the classification category increased from 10 to 101 but also the number of categories has increased 10 times. Moreover, the number of images available for training has been reduced from 60000 to 9000, which is a great challenge to the convolutional neural network model. Generally speaking, the accuracy of image classification will be significantly reduced, and the difference between models will be more obvious.  Table 2 supports this conjecture.

The data obtained in Table 2 are the results of the training Caltech-101 training set with 50 epochs. Due to the increasing difficulty of image classification in the Caltech-101 dataset, the classification accuracy of the test set is significantly lower than that of the Cifar-10 dataset. It can be found that the accuracy of the five models on the Caltech-101 test set is about 60%–70%, and the difference is also highlighted. At the same time, it can also be found that the convolution neural network can fit the training set of the dataset well, and the accuracy of the model training set is more than 98%, but the accuracy of the test set is different. AA-ResNet has also reached about 70%, which is the most accurate of several models. Moreover, due to the reduction in the number of images, the training time of a single epoch decreased significantly, from 253.71 seconds to 55.82 seconds, the number of training images decreased 3 times, and the training time decreased 4 times.

**Figure 5:**
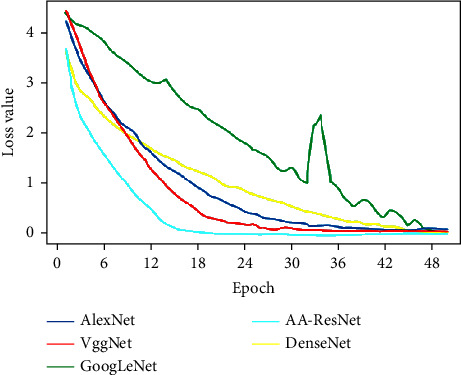
Loss value of Caltech-101 data set.

In Figure 5, the starting position of the loss value of the five models is near 4, which is higher than that of the previous Cifar-10 experiment. Among them, the loss value of AA-ResNet is around 0 at 14 epochs, which is far ahead of the other models. This also proves that the performance of the convolutional neural network model in the Caltech-101 data set is not as good as that in the Cifar-10 data set, because there are not many bright blocks outside the diagonal in the confusion matrix of Cifar-10, and there are no black blocks on the diagonal. At the same time, it can also be found from the confusion matrix that the classification effect of each model on small classes is inconsistent, and the classes with good and poor image classification effects of each model are inconsistent, which also shows the differences between models.

**Figure 6:**
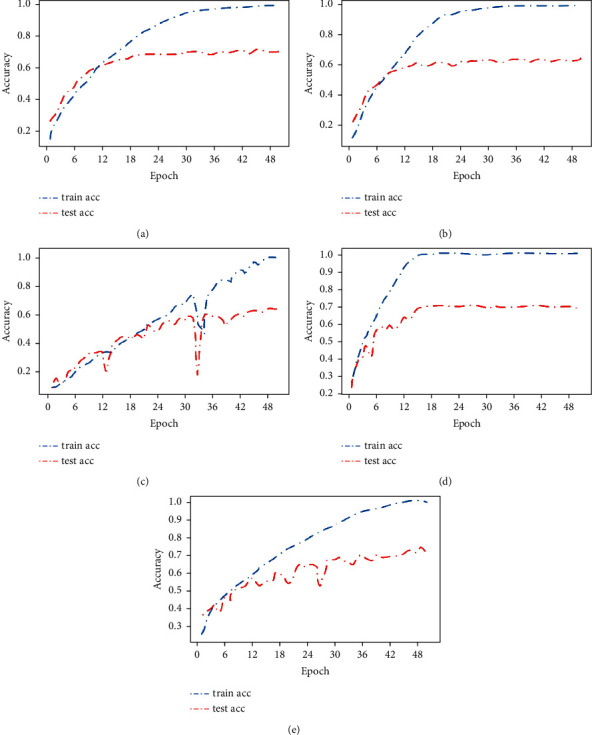
Accuracy rate of Caltech-101 data set. (a) AlexNet. (b) VggNet. (c) GoogLeNet. (d) AA-ResNet. (e) DenseNet.

As shown in Figure 6, the Caltech-101 data set takes 50 epochs to make all models fit well, and the training set accuracy rate train acc reaches more than 95%. The corresponding Cifar-10 data set only takes 30 epochs, which also proves that the Caltech-101 data set is more complex and difficult to fit. It can be seen from the figure that AA-ResNet has a fast fitting speed and the accuracy rate of the training set is close to 100% when it is around 20 epochs. However, the GoogLeNet and DenseNet models fluctuate greatly. Even GoogLeNet has a large “*V*” shaped fluctuation, which is generally caused by a poor fitting effect. It can also be found that when the training set accuracy rate train acc and the test set accuracy rate test acc curves tend to be stable. The two lines are separated by a large distance, which also indicates that the model has not achieved a particularly good effect.

On the whole, it can also be found that Caltech-101 is more complex. The fitting effect of the model is lower than that of the previous Cifar-10 experimental model, and it shows differences. AA-ResNet performs well as a whole, with small fluctuations and fast fitting speed.

### 4.6. Caltech-256 Data Set Experimental Results

The Caltech-256 data set is much more complex than the Cifar-10 and Caltech-101 data sets in the above experiments. It is also a data set that is difficult to classify in all image classification data sets. The Caltech-256 dataset is composed of 256 categories, with a large number of categories, and the images in the dataset differ greatly in object size and position. All this means that the difficulty of classification is increased, to explore the specific performance of each network model for this complex situation and the differences in the complex situation.  Table 3 shows the experimental results of the Caltech-256 data set.

The data in Table 3 are the results of the Caltech-256 training set with 50 epochs. It can be found that the accuracy rate is significantly lower than that of the above Cifar-10 and Caltech-101 data set experiments. The accuracy rate of the test sets of the five models is within the range of 30%–55%, and the difference between the models is greater. When the number of epochs trained in the Caltech-101 experiment is the same, the accuracy rate of the five model testing sets is lower, of which the accuracy rate of AA-ResNet is about 50%. Since the number of images in the Caltech-256 dataset is three times that of the Caltech-101, the training time is also greatly increased. It can be seen that the training time is in direct proportion to the number of images.

**Figure 7:**
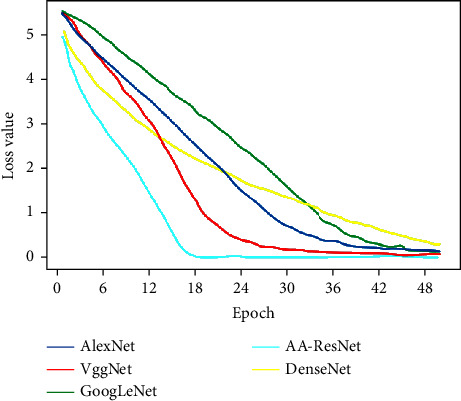
Loss value of Caltech-256 data set.

In Figure 7, the loss value of the five models gradually decreases from about 5 to about 0 with the increase in the epoch number. AA-ResNet first approaches 0 at 18 epochs, which indicates that AA-ResNet is ahead of the other models. The diagonal line of the AA-ResNet confusion matrix is brighter, so it is analyzed that the image classification effect of this model is better.

**Figure 8:**
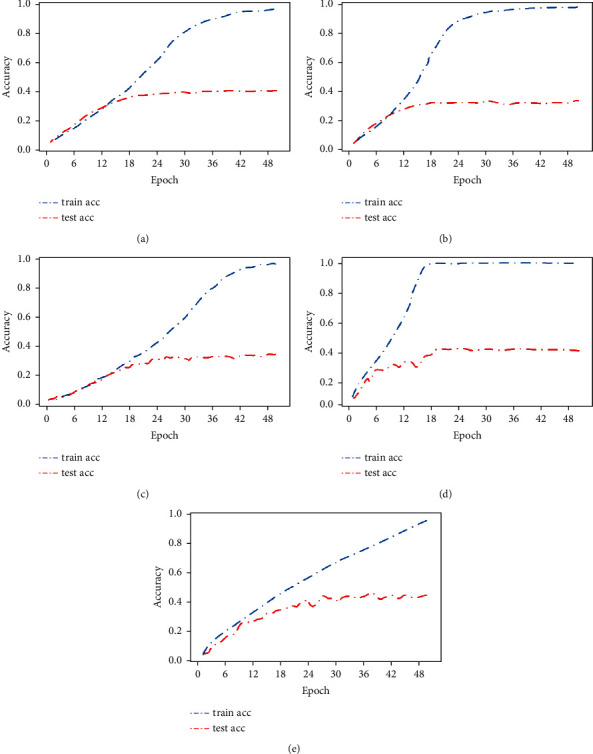
Accuracy rate of Caltech-256 data set. (a) AlexNet. (b) VggNet. (c) GoogLeNet. (d) AA-ResNet. (e) DenseNet.

As shown in Figure 8, the accuracy starting points of the five models are all around 0%. This phenomenon is caused by the complexity of the Caltech-256 data set. Consistent with the above experiments on Cifar-10 and Caltech-101 data sets, the fitting speed of AA-ResNet is the fastest among the five models, and the fitting is completed in about 18 epochs, and the accuracy of the training set is nearly 100%. What is inconsistent with the above experiment is that the fluctuation of the model is relatively small, and the distance between the training set accuracy train acc curve and the test set accuracy test acc curve is larger. It can be seen that Caltech-256 is extremely complex and the fitting effect of the model is poor, but it shows a greater difference. In general, among the five models, the AA-ResNet proposed in this paper performs well. The test set has the highest accuracy, and the classification effect of each class is better.

## 5. Conclusions and Future Work

In the experiment, we use the data sets of Cifar-10, Caltech-101, and Caltech-256 to test the superiority of the proposed AA-ResNet model compared with the AlexNet, VggNet, GoogLeNet, and DenseNet models, and verify that AA-ResNet has better performance in image classification. By comparing the training time, accuracy and loss value parameters of the five models and drawing the confusion matrix, accuracy, and loss value change line graph, it is found that AA-ResNet has the best performance among the five convolutional neural network models, and the fitting speed of AA-ResNet is faster.

In the future, we can continue to improve the accuracy of the model by increasing the layers of AA-ResNet and spending more time to adjust the parameters of the model. Due to the limitations of the hardware and software, the relevant data for the complete model will be supplemented in the subsequent experiments. Although the convolution neural network model is applied to image classification and recognition in this paper, the running state of the relevant model on the server is not evaluated. A convolutional neural network model can not only be applied in the field of image recognition but also plays an important role in natural language processing. It needs further theoretical research and practical application.

## Figures and Tables

**Table 1:** Experimental results of Cifar-10 data set.

	AlexNet	VggNet	GoogLeNet	AA-ResNet	DenseNet
Train Acc	98.58%	98.93%	99.67%	99.96%	98.78%
Test Acc	91.42%	87.85%	90.54%	92.43%	91.11%
Loss	0.04569	0.03547	0.11288	0.00129	0.03543
Time (s)	145.71	202.68	306.90	253.71	374.37

**Table 2:** Experimental results of Caltech-101 data set.

	AlexNet	VggNet	GoogLeNet	AA-ResNet	DenseNet
Train Acc	98.45%	99.29%	99.35%	99.96%	99.81%
Test Acc	70.07%	63.74%	63.32%	69.61%	71.28%
Loss	0.05628	0.03123	0.01042	0.00352	0.03112
Time (s)	42.55	43.56	61.27	55.82	70.91

**Table 3:** Experimental results of Caltech-256 data set.

	AlexNet	VggNet	GoogLeNet	AA-ResNet	DenseNet
Train Acc	96.28%	98.47%	96.34%	99.88%	93.94%
Test Acc	40.13%	33.21%	35.67%	52.29%	47.26%
Loss	0.12786	0.04983	0.14234	0.00217	0.28646
Time (s)	152.34	176.92	209.25	211.46	263.55

## Data Availability

The authors confirm that the data supporting the findings of this study are available within the article.
